# Body mass index, abdominal fatness, weight gain and the risk of psoriasis: a systematic review and dose–response meta-analysis of prospective studies

**DOI:** 10.1007/s10654-018-0366-z

**Published:** 2018-04-21

**Authors:** Dagfinn Aune, Ingrid Snekvik, Sabrina Schlesinger, Teresa Norat, Elio Riboli, Lars J. Vatten

**Affiliations:** 1 Department of Epidemiology and Biostatistics, School of Public Health, Imperial College London, St. Mary’s Campus, Norfolk Place, Paddington, London, W2 1PG UK; 2 Department of Nutrition, Bjørknes University College, Oslo, Norway; 3 Department of Endocrinology, Morbid Obesity and Preventive Medicine, Oslo University Hospital, Oslo, Norway; 4 Department of Public Health and Nursing, Faculty of Medicine, Norwegian University of Science and Technology, Trondheim, Norway; 5 Department of Dermatology, St. Olavs Hospital, Trondheim University Hospital, Trondheim, Norway; 6 German Diabetes Center (DDZ), Institute for Biometrics and Epidemiology, Heinrich Heine University Düsseldorf, Düsseldorf, Germany

**Keywords:** Body mass index, Waist circumference, Waist-to-hip ratio, Weight gain, Psoriasis, Systematic review, Meta-analysis

## Abstract

Greater body mass index (BMI) has been associated with increased risk of psoriasis in case–control and cross-sectional studies, however, the evidence from prospective studies has been limited. We conducted a systematic review and dose–response meta-analysis of different adiposity measures and the risk of psoriasis to provide a more robust summary of the evidence based on data from prospective studies. PubMed and Embase databases were searched for relevant studies up to August 8th 2017. Summary relative risks (RRs) and 95% confidence intervals (CIs) were calculated using a random effects model. The summary relative risk (RR) for a 5 unit increment in BMI was 1.19 (95% CI 1.10–1.28, I^2^ = 83%, n = 7). The association appeared to be stronger at higher compared to lower levels of BMI, *p*_nonlinearity_ < 0.0001, and the lowest risk was observed at a BMI around 20. The summary RR was 1.24 (95% CI 1.17–1.31, I^2^ = 0%, *p*_heterogeneity_ = 0.72, n = 3) per 10 cm increase in waist circumference, 1.37 (95% CI 1.23–1.53, I^2^ = 0%, *p*_heterogeneity_ = 0.93, n = 3) per 0.1 unit increase in waist-to-hip ratio, and 1.11 (95% CI 1.07–1.16, I^2^ = 47%, *p*_heterogeneity_ = 0.15, n = 3) per 5 kg of weight gain. Adiposity as measured by BMI, waist circumference, waist-to-hip ratio, and weight gain is associated with increased risk of psoriasis.

**Electronic supplementary material:**

The online version of this article (10.1007/s10654-018-0366-z) contains supplementary material, which is available to authorized users.

## Introduction

Psoriasis is a chronic immune-mediated inflammatory skin disease which is characterized by patches of abnormal skin, which typically are red, itchy and scaly [1]. The condition affects approximately 2% of the general population, however, the prevalence has doubled over the recent decades in some countries, reaching a lifetime prevalence of 5.8–11% in Scandinavia [2, 3]. A diagnosis of psoriasis has been associated with increased risk of coronary heart disease [4, 5], atrial fibrillation [6], stroke [5–7], thromboembolism [7], certain cancers [8], and possibly other conditions [9–13]. Although several risk factors for psoriasis have been suggested or established including age [14], family history of psoriasis [15, 16], skin infections and skin disorders [17], gallstones [18], smoking [19], alcohol consumption [20], and physical inactivity [21, 22], much remains to be learned.

Adiposity is associated with low-grade inflammation through overproduction of inflammatory cytokines. Activated macrophages in adipose tissue stimulate adipocytes to secrete TNF-α, IL-1, IL-6, and IL-8, which may contribute to the development of psoriasis [23, 24]. In addition, higher levels of leptin, an adipokine related to obesity, has also been associated with increased risk of psoriasis [25–27]. A large number of cross-sectional and case–control studies have reported a positive association between adiposity and the risk of psoriasis [28–42]. A meta-analysis from 2012 also suggested a positive association between obesity (BMI of ≥ 30 kg/m^2^) and psoriasis [43], however, it was largely based on cross-sectional and case–control studies, study designs which can be difficult to rely on for causal inferences because (1) the temporality of the association between the exposure and the disease is not clear in cross-sectional studies, and (2) because case–control studies may be hampered by recall and selection biases. However, recently several prospective studies have also reported on the association between body mass index (BMI, kg/m^2^), abdominal fatness (waist circumference or waist-to-hip ratio) or weight changes and the risk of psoriasis [17, 44–49]. A large study from the UK found a 33% increase in the risk of psoriasis among obese participants compared to normal weight (BMI of 18.5 to  < 25.0 kg/m^2^) participants [17], while in the Nurses’ Health Study I and II there was roughly a doubling in the relative risk among those with grade 2 obesity (BMI of ≥ 35 kg/m^2^) compared to those with normal weight [44, 45]. Furthermore there was a doubling in the risk among obese participants compared to normal weight participants in the Danish National Birth Cohort [46]. In contrast, a large Korean study only found a weak and non-statistically significant association [48] and a Norwegian study did not find a significant association with overweight or obesity [47], while a second much larger Norwegian study found an almost twofold increase in risk of psoriasis among obese persons [49]. The lower BMI in the Korean population compared to the European and American populations may have contributed to the weaker association in that study. With regard to overweight, the results have been less consistent with three studies reporting statistically significant positive associations [44, 45, 49], while other studies found no clear association [17, 46, 47, 48]. Abdominal fatness (waist circumference and waist-to-hip ratio) is a better predictor of visceral fat than BMI [50], and may be more strongly associated with insulin resistance than BMI [51], however, it is unclear whether waist circumference or waist-to-hip ratio is more strongly associated with psoriasis than BMI. The few studies that investigated the association between waist circumference, waist-to-hip ratio and weight gain were consistent in reporting an increased risk with increasing adiposity [44, 45, 49]. Given the limited number of prospective studies and lack of data on abdominal fatness in previous meta-analyses on adiposity and psoriasis we therefore conducted a systematic review and dose–response meta-analysis of prospective studies to clarify the strength and shape of the dose–response relationship between different measures of adiposity and the risk of psoriasis.

## Methods

### Search strategy

We searched the PubMed and the Embase databases up to August 8th 2017. The search terms used are found in the Supplementary Text. We followed the PRISMA criteria for the reporting of meta-analyses of observational studies [52]. In addition, the reference lists of all the studies that were included in the analysis and the reference list of a published meta-analysis were searched for additional studies [43]. DA and SS conducted the screening of the literature search.

### Study selection

Prospective or retrospective cohort studies, case-cohort studies, or nested case–control studies of the association between measures of body fatness and risk of psoriasis were included. Relative risk (RR) estimates (hazard ratio, risk ratio, odds ratio) had to be available with the 95% confidence intervals (CIs) in the publication and for the dose–response analysis, a quantitative measure of the exposure and the total number of cases and person-years had to be available in the publication. If there were several publications from the same study we used the study with the largest number of cases, or the study which provided sufficient detail of data to be included in dose–response analyses. A list of the excluded studies and exclusion reasons is found in Supplementary Table 1.

### Data extraction

We extracted from each study: The first author’s last name, publication year, country where the study was conducted, the name of the cohort, follow-up period, sample size, sex, age, number of cases, assessment method of anthropometric factors (measured vs. self-reported), type of anthropometric measure, RRs and 95% CIs, and variables adjusted for in the analysis. Data were extracted by DA and checked for accuracy by SS.

### Statistical analysis

Summary RRs and 95% CIs for a 5 unit increment in BMI, 5 kg increase in weight gain, 10 cm increment in waist circumference, and for a 0.1 unit increment in waist-to-hip ratio (consistent with our previous analyses [53, 54]) were estimated using a random effects model [55]. The average of the natural logarithm of the RRs was estimated and the RR from each study was weighted using random effects weights [55]. A two-tailed *p* < 0.05 was considered statistically significant.

The method described by Greenland and Longnecker [56] was used for the dose–response analysis and study-specific slopes (linear trends) and 95% CIs were computed from the natural logs of the RRs and CIs across categories of adiposity measures. The method requires that the distribution of cases and person-years or non-cases and the RRs with the variance estimates for at least three quantitative exposure categories are known. We estimated the distribution of cases or person-years in studies that did not report these, but reported the total number of cases and person-years [53]. The mean level of BMI, waist circumference, waist-to-hip ratio, and weight gain in each category was assigned to the corresponding relative risk for each study and for studies that reported these measures by ranges, we estimated the midpoint in each category as the average of the lower and upper bounds. For studies which did not use the lowest category as the reference category we converted the risk estimates so that the lowest category became the reference category using the method by Hamling [57]. A potential nonlinear dose–response relationship between BMI, waist circumference, waist-to-hip ratio, weight gain and psoriasis was examined by using fractional polynomial models [58]. We determined the best fitting second order fractional polynomial regression model, defined as the one with the lowest deviance. A likelihood ratio test was used to assess the difference between the nonlinear and linear models to test for nonlinearity [58]. Study quality was assessed using the Newcastle–Ottawa scale which rates studies according to selection, comparability and outcome assessment with a score range from 0 to 9 [59].

Subgroup and meta-regression analyses were conducted to investigate potential sources of heterogeneity including study characteristics such as sex, duration of follow-up, geographic location, number of cases, study quality and adjustment for confounding factors. Heterogeneity between studies was quantitatively assessed by the Q test and I^2^ [60]. Small study effects, such as publication bias, were assessed by inspecting the funnel plots for asymmetry and with Egger’s test [61] and Begg’s test [62], with the results considered to indicate small study effects when *p* < 0.10. Sensitivity analyses excluding one study at a time were conducted to clarify whether the results were simply due to one large study or a study with an extreme result.

## Results

**Fig. 1:**
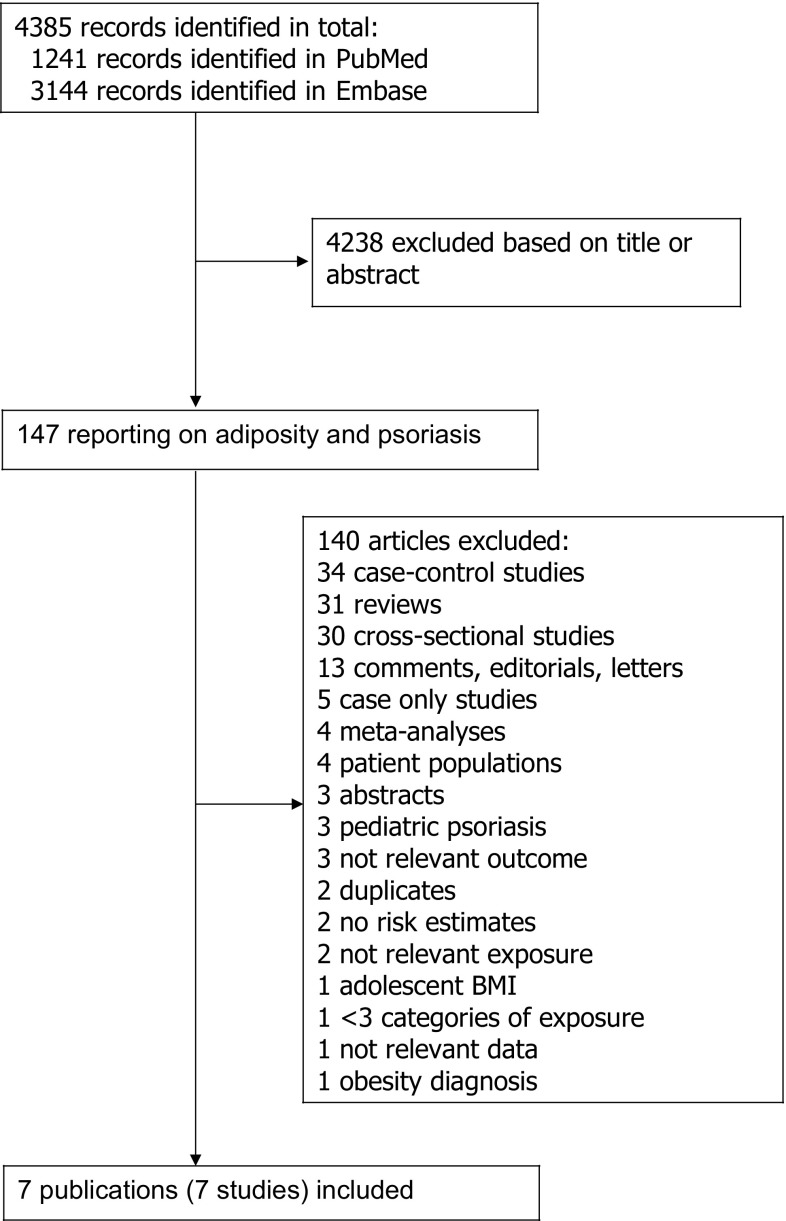
Flow-chart of study selection

We identified 7 prospective studies (7 publications) [17, 44–49] that were included in the analyses of adiposity and psoriasis risk (Table 1, Fig. 1). Characteristics of the included studies are provided in Table 1. Four studies were from Europe, two studies were from the US, and one study was from Asia (Table 1).

**Table 1:** Prospective studies of adiposity and risk of psoriasis

References, country	Study name	Follow-up period	Study size, sex, age, number of cases	Assessment of weight and height	Study quality score	Exposure and subgroup	Description of quantiles or categories	RR (95% CI)	Adjustment for confounders
Huerta et al., 2007, United Kingdom [17]	UK General Practitioners Database	1996–1997, ~ 1 year follow-up	Nested case–control study: 3994 cases 10,000 controls All ages	Measured	7	BMI	< 20	0.99 (0.84–1.17)	Age, sex, calendar year, smoking, visits to GP in the last year
							20–24	1.00	
							25–29	1.11 (1.00–1.24)	
							≥ 30	1.33 (1.16–1.52)	
Setty et al., 2007, USA [44]	Nurses’ Health Study II	1991–2005, 14 years follow-up	78,626 women, age 25–42 years: 892 cases 890 cases (BMI, baseline) 884 cases (BMI at age 18 years) 336 cases (waist circumference) 334 cases (hip circumference, waist-to-hip ratio) 838 cases (weight change)	Self-reported, validated	7	BMI, updated	< 21 21–22.9 23–24.9 25–29.9 30–34.9 ≥ 35	0.81 (0.63–1.06) 1 1.19 (0.94–1.51) 1.40 (1.13–1.73) 1.48 (1.15–1.91) 2.69 (2.12–3.40)	Age, alcohol, smoking status
						BMI, baseline	< 21	0.80 (0.65–0.99)	
							21–22.9	1	
							23–24.9	1.14 (0.92–1.41)	
							25–29.9	1.23 (1.00–1.50)	
							30–34.9	1.73 (1.36–2.20)	
							≥ 35	2.23 (1.72–2.87)	
						BMI at age 18 years	< 21	0.76 (0.65–0.90)	
							21–22.9	1	
							23–24.9	1.02 (0.81–1.28)	
							25–29.9	0.97 (0.75–1.25)	
							≥ 30	1.73 (1.24–2.41)	
						Waist circumference	< 31 in.	1	
							31–33.9	1.32 (0.99–1.77)	
							34–36.9	1.59 (1.14–2.21)	
							37–40.0	1.73 (1.18–2.53)	
							>40	2.28 (1.57–3.32)	
						Hip circumference	36 in.	1	
							36.1–37.9	1.18 (0.79–1.75)	
							38.0–39.4	1.45 (1.01–2.10)	
							39.5–42.0	1.42 (0.99–2.05)	
							>42.0	2.22 (1.57–3.12)	
						Waist-to-hip ratio	0.47–0.72	1	
							0.73–0.75	0.94 (0.65–1.36)	
							0.76–0.78	1.01 (0.70–1.45)	
							0.79–0.83	1.16 (0.83–1.63)	
							0.84–1.56	1.57 (1.13–2.17)	
						Weight change between age 18 years and baseline	− 5.0 lb	0.85 (0.61–1.17)	
							− 5.0 to + 4.9	1	
							+ 5.0 to + 19.9	1.00 (0.80–1.25)	
							+ 20 to + 34.9	1.12 (0.88–1.43)	
							+ 35	1.54 (1.22–1.94)	
						Weight change between age 18 years and updated follow-up	− 5.0 lb	0.91 (0.60–1.37)	
							− 5.0 to + 4.9	1	
							+ 5.0 to + 19.9	1.24 (0.93–1.64)	
							+ 20 to + 34.9	1.35 (1.01–1.80)	
							+ 35	1.88 (1.44–2.46)	
Kumar et al. 2013, USA [45]	Nurses’ Health Study 1	1996–2008, 12 years follow-up	67,300 women, mean age 62 years: 809 cases	Self-reported, validated	7	BMI, baseline	18.5–24.9	1	Age, alcohol, smoking status, physical activity
							25–29.9	1.11 (0.94–1.31)	
							30–34.9	1.71 (1.40–2.08)	
							≥ 35	1.63 (1.24–2.14)	
			722 cases (weight change) 675 cases (waist circumference, waist-to-hip ratio)				Per 1 unit	1.04 (1.02–1.05)	
						BMI, updated	18.5–24.9	1	
							25–29.9	1.21 (1.03–1.43)	
							30–34.9	1.63 (1.33–2.00)	
							≥ 35	2.03 (1.58–2.61)	
							Per 1 unit	1.04 (1.03–1.05)	
						Waist circumference	20–28 in.	1	
							29–32	1.06 (0.87–1.29)	
							33–66	1.50 (1.24–1.82)	
							Per 1 SD	1.20 (1.11–1.29)	
						Waist-to-hip ratio	0.41–0.75	1	
							0.75–0.80	1.25 (1.03–1.51)	
							0.80–2.1	1.40 (1.15–1.70)	
							Per 1 SD	1.09 (1.02–1.16)	
						Weight change	− < 5 lbs	1.06 (0.69–1.64)	Weight change also adjusted for BMI at 18 years
							− 5 to + 4.9	1	
							+ 5 to + 19.9	1.16 (0.81–1.64)	
							+ 20 to + 34.9	1.40 (0.99–1.96)	
							+ 35	1.79 (1.29–2.47)	
							Per 10 lbs	1.08 (1.06–1.11)	
Harpsøe et al. 2014, Denmark [46]	Danish National Birth Cohort	1996–2002–2011, 11 years follow-up	75,008 women, median age 30.2 years: 109 cases	Self-reported	7	BMI	< 18.5	0.91 (0.33–2.51)	Age, smoking, alcohol, parity, socioeconomic status
							18.5 to < 25	1	
							25 to < 30	1.38 (0.87–2.20)	
							**≥**30	2.16 (1.25–3.72)	
							Per 1 unit	1.03 (0.99–1.07)	
Danielsen et al. 2016, Norway [47]	Tromsø Study	1994–2008, 7–13 years follow-up	8752 men and women, age 25–69 years: 409 cases	Measured	7	BMI	< 25 25 to < 30 ≥ 30 Per 2.5 units	1 1.00 (0.80–1.25) 1.35 (0.97–1.87) 1.08 (1.01–1.16)	Age, sex (combined analysis), current smoking, daily alcohol intake, recreational physical activity score
						BMI, men	< 25	1	
							25 to < 30	0.96 (0.70–1.31)	
							≥ 30	1.24 (0.76–2.03)	
							Per 2.5 units	1.10 (0.98–1.23)	
						BMI, women	<25	1	
							25 to < 30	1.05 (0.76–1.45)	
							≥ 30	1.48 (0.94–2.31)	
							Per 2.5 units	1.07 (0.98–1.17)	
						BMI change, < 45 years age	1	1	
							2	0.96 (0.59–1.57)	
							3	0.89 (0.54–1.48)	
							4	0.79 (0.46–1.35)	
						BMI change, ≥ 45 years age	1	1	
							2	1.25 (0.85–1.84)	
							3	1.44 (0.97–2.12)	
							4	1.70 (1.13–2.55)	
Kim et al. 2017, Korea [48]	Korea National Health Insurance	2002–2013, 8.5 years follow-up	418,057 men and women, age ≥ 20 years: 11,054 cases	Measured	8	BMI	< 18.5	0.92 (0.83–1.03)	Age, sex, smoking, alcohol, exercise, income
							18.5–22.9	1	
							23.0–24.9	1.01 (0.96–1.06)	
							≥ 25.0	1.05 (1.00–1.10)	
Snekvik et al. 2017, Norway [49]	The HUNT Study 2–3 (and HUNT 1 for weight change)	1984–1986, 1995–1997–2006–2008, ~ 11.1 years follow-up	33,734 men and women, age ≥ 20 years: 369 cases 33,485/33,485/25,148 participants for analyses of waist circumference, waist-to-hip ratio, and weight change: 367/367/267 cases	Measured	8	BMI, all	18.5–24.9 25.0–29.9 ≥ 30.0 Per 3.81 units Per 1 unit	1 1.45 (1.15–1.84) 1.87 (1.38–2.52) 1.22 (1.11–1.34) 1.05 (1.03–1.08)	Age, sex, education, smoking status
						Waist circumference	≤ 86/≤ 73 cm (m/f)	1	
							87–90/74–79	1.33 (0.98–1.81)	
							91–96/80–86	1.48 (1.09–2.00)	
							≥ 97/≥ 87	1.95 (1.46–2.61)	
							Per 11.14 cm	1.26 (1.15–1.39)	
							Per 1 cm	1.02 (1.01–1.03)	
						Waist-to-hip ratio	≤ 0.86/≤ 0.75 (m/f)	1	
							0.86–0.89/0.75–0.79	1.19 (0.88–1.61)	
							0.89–0.92/0.79–0.82	1.13 (0.83–1.54)	
							≥ 0.92/≥ 0.82	1.53 (1.14–2.07)	
							Per 0.08 unit	1.18 (1.07–1.31)	
							Per 0.1 unit	1.35 (1.12–1.61)	
						Weight change	− 2.0 kg	0.66 (0.32–1.33)	
							− 2.0 to + 1.9 kg	1	
							2.0–4.9	1.05 (0.69–1.61)	
							5.0–9.9	1.33 (0.90–1.95)	
							≥ 10.0	1.72 (1.15–2.58)	
							Per 5.96 kg	1.20 (1.07–1.35)	
							Per 1 kg	1.03 (1.01–1.05)	
						BMI, men	18.5–24.9	1	
							25.0–29.9	1.75 (1.22–2.51)	
							≥ 30.0	2.46 (1.57–3.85)	
							Per 3.81 units	1.28 (1.12–1.45)	
							Per 1 unit	1.08 (1.04–1.12)	
						Waist circumference	≤ 86/≤ 73 cm (m/f)	1	
							87–90/74–79	1.38 (0.86–2.20)	
							91–96/80–86	1.73 (1.11–2.68)	
							≥ 97/≥ 87	2.43 (1.59–3.70)	
							Per 11.14 cm	1.36 (1.19–1.54)	
							Per 1 cm	1.04 (1.02–1.05)	
						Waist-to-hip ratio	≤ 0.86/≤ 0.75 (m/f)	1	
							0.86–0.89/0.75–0.79	1.30 (0.84–2.02)	
							0.89–0.92/0.79–0.82	1.28 (0.82–2.01)	
							≥ 0.92/≥ 0.82	1.89 (1.23–2.92)	
							Per 0.08 unit	1.31 (1.14–1.51)	
							Per 0.1 unit	1.66 (1.27–2.17)	
						Weight change	− 2.0 kg	0.77 (0.28–2.08)	
							− 2.0 to + 1.9 kg	1	
							2.0–4.9	1.17 (0.63–2.17)	
							5.0–9.9	1.62 (0.92–2.85)	
							≥ 10.0	2.15 (1.18–3.91)	
							Per 5.96 kg	1.30 (1.09–1.55)	
							Per 1 kg	1.04 (1.01–1.08)	
						BMI, women	18.5–24.9	1	
							25.0–29.9	1.26 (0.91–1.74)	
							≥ 30.0	1.51 (0.99–2.29)	
							Per 3.81 units	1.17 (1.02–1.34)	
							Per 1 unit	1.04 (1.00–1.07)	
						Waist circumference	≤ 86/≤ 73 cm (m/f)	1	
							87–90/74–79	1.29 (0.85–1.95)	
							91–96/80–86	1.27 (0.82–1.95)	
							≥ 97/≥ 87	1.57 (1.04–2.37)	
							Per 11.14 cm	1.17 (1.01–1.34)	
							Per 1 cm	1.01 (1.00–1.03)	
						Waist-to-hip ratio	≤ 0.86/≤ 0.75 (m/f)	1	
							0.86–0.89/0.75–0.79	1.11 (0.73–1.68)	
							0.89–0.92/0.79–0.82	1.01 (0.66–1.55)	
							≥ 0.92/≥ 0.82	1.26 (0.83–1.92)	
							Per 0.08 unit	1.08 (0.93–1.25)	
							Per 0.1 unit	1.14 (0.88–1.47)	
						Weight change	− 2.0 kg	0.57 (0.22–1.53)	
							− 2.0 to + 1.9 kg	1	
							2.0–4.9	0.95 (0.53–1.70)	
							5.0–9.9	1.07 (0.63–1.83)	
							≥ 10.0	1.42 (0.82–2.44)	
							Per 5.96 kg	1.14 (0.97–1.33)	
							Per 1 kg	1.02 (0.99–1.05)

### Body mass index

**Fig. 2:**
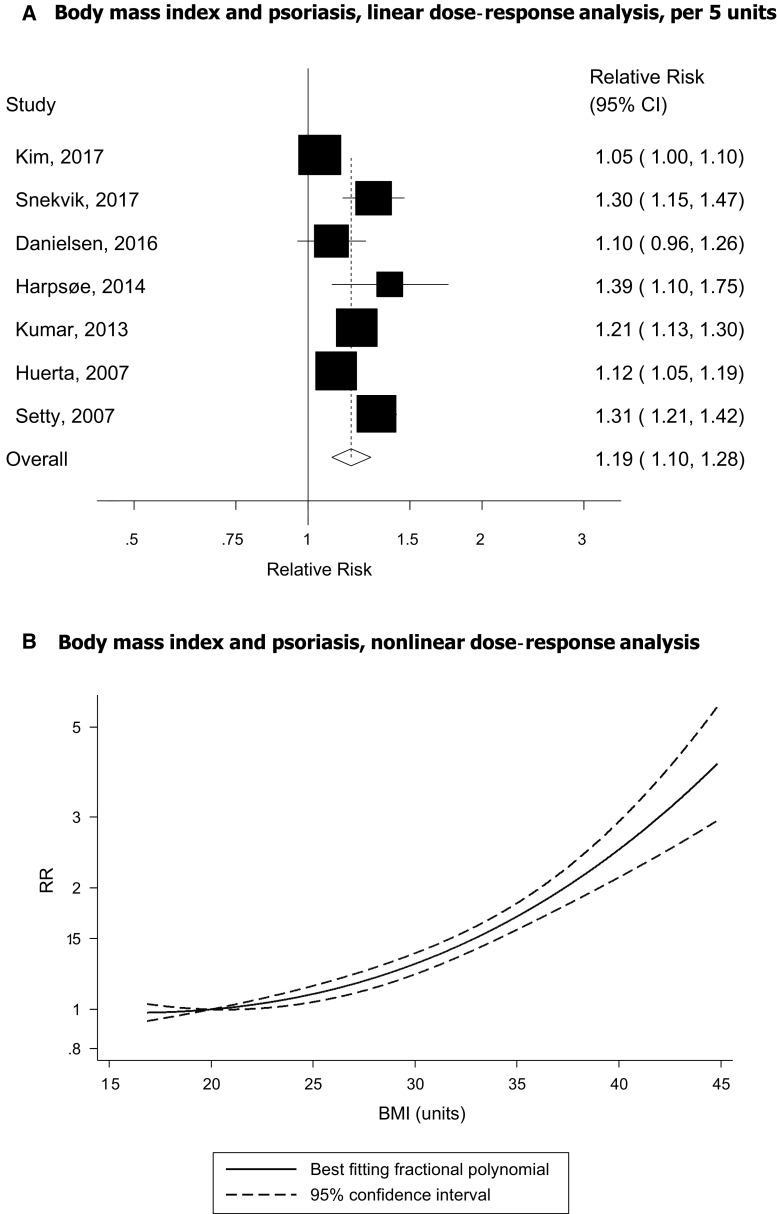
BMI and psoriasis

Seven prospective studies [17, 44–49] were included in the analysis of BMI and psoriasis risk including 17,636 cases and 695 471 participants. The summary relative risk (RR) for a 5 unit increment was 1.19 (95% CI 1.10–1.28, I^2^ = 83.1%, *p*_heterogeneity_ < 0.0001) (Fig. 2a). There was no evidence of publication bias with Egger’s test, *p* = 0.12, or Begg’s test, *p* = 0.37 (Supplementary Fig. 1). In sensitivity analyses, the summary RR ranged from 1.16 (95% CI 1.08–1.25) when excluding the Nurses’ Health Study II [44] to 1.22 (95% CI 1.14–1.30) when excluding the Korea National Health Insurance Corporation study [48]. The summary RR was 1.25 (95% CI 1.19–1.31, I^2^ = 1%, n = 5) for women and 1.34 (95% CI 1.11–1.62, I^2^ = 35%, n = 2) for men (Supplementary Table 2). There was evidence of a nonlinear association between BMI and psoriasis, *p*_nonlinearity_ < 0.0001, and there was a steeper increase in risk at higher compared to lower levels of BMI, however, some evidence of increased risk was observed even within the normal BMI range and the lowest risk was observed with a BMI between 16.75 and 20 (Fig. 2b, Supplementary Table 3).

### Waist circumference

**Fig. 3:**
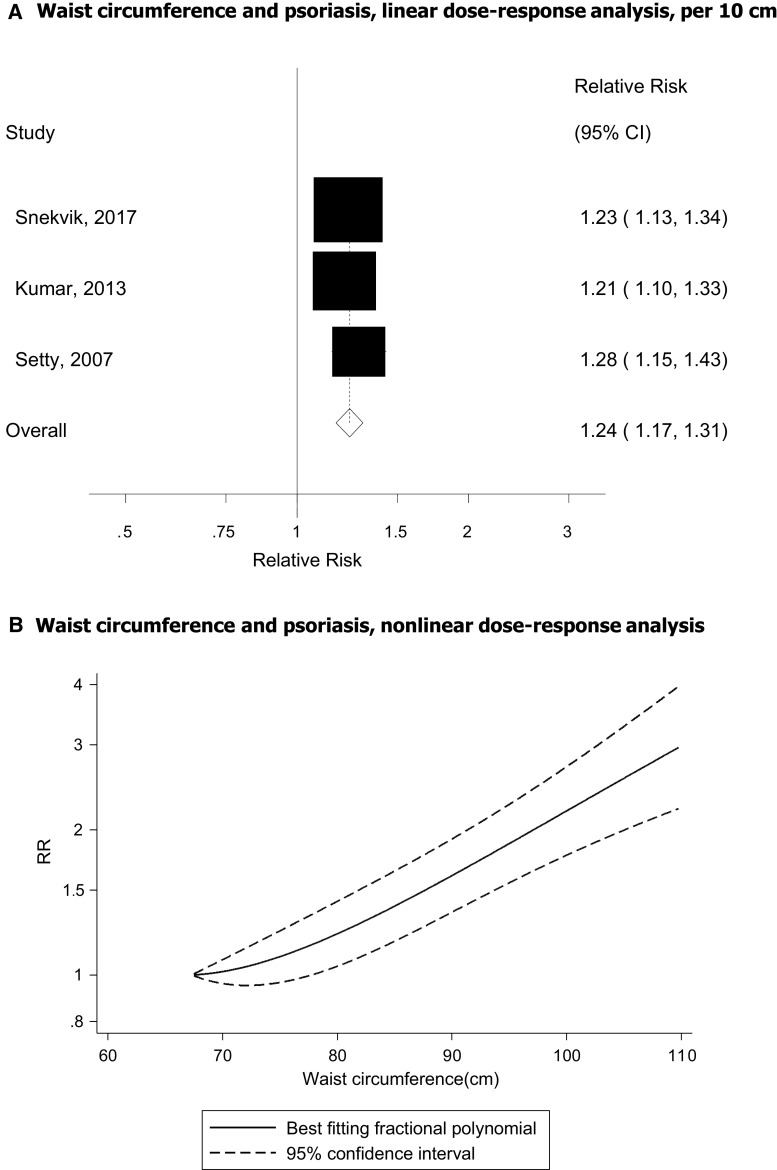
Waist circumference and psoriasis

Three cohort studies [44, 45, 49] were included in the analysis of waist circumference and psoriasis risk and included 2068 cases among 179 411 participants. The summary RR was 1.24 (95% CI 1.17–1.31, I^2^ = 0%, *p*_heterogeneity_ = 0.72) per 10 cm increase in waist circumference (Fig. 3a). The summary RR was 1.22 (95% CI 1.15–1.30, I^2^ = 0%, n = 3) for women and 1.44 (95% CI 1.23–1.66, n = 1) for men. There was no evidence of a nonlinear association between waist circumference and psoriasis risk, *p*_nonlinearity_ = 0.09 (Fig. 3b, Supplementary Table 4).

### Waist-to-hip ratio

**Fig. 4:**
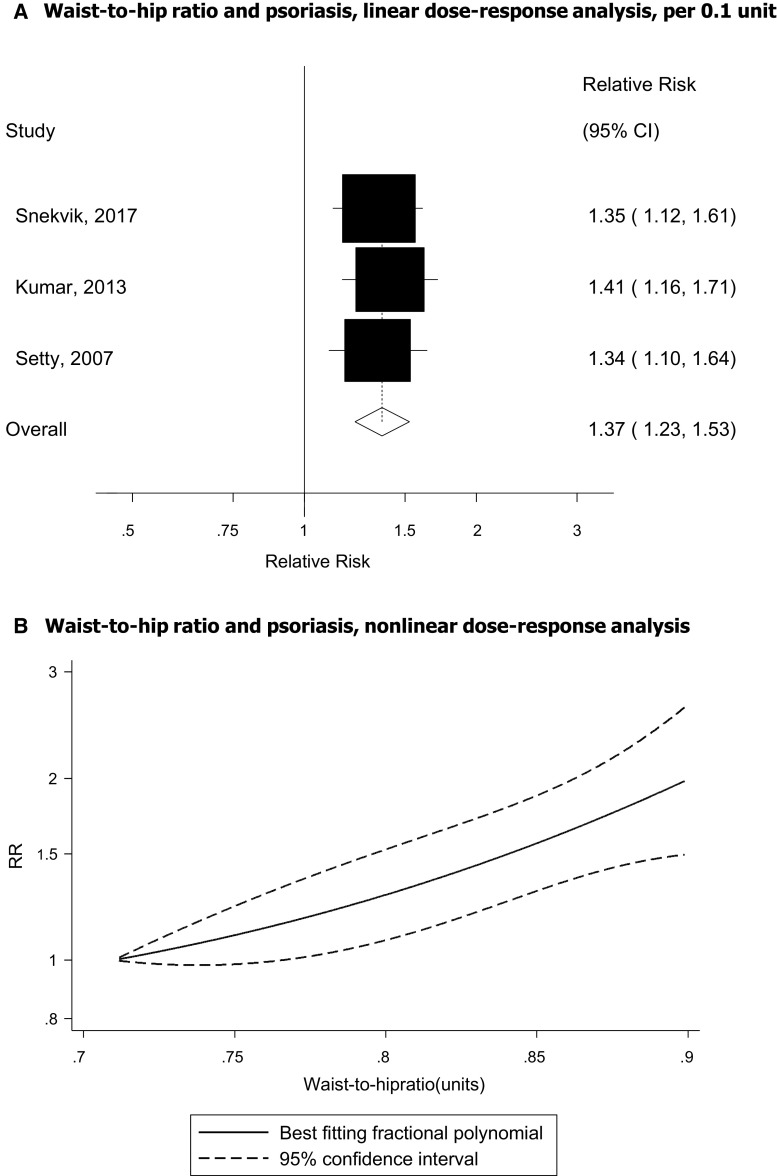
Waist-to-hip ratio and psoriasis

Three cohort studies [44, 45, 49] were included in the analysis of waist-to-hip ratio and psoriasis risk and included 2068 cases among 179 411 participants. The summary RR for a 0.1 unit increment in waist-to-hip ratio was 1.37 (95% CI 1.23–1.53, I^2^ = 0%, *p*_heterogeneity_ = 0.93) per 0.1 unit increase in waist-to-hip ratio (Fig. 4a). The summary RR was 1.32 (95% CI 1.17–1.49, I^2^ = 0%, n = 3) for women and 1.31 (95% CI 1.14–1.51, n = 1) for men. There was no evidence of a nonlinear association between waist-to-hip ratio and psoriasis risk, *p*_nonlinearity_ = 0.59 (Fig. 4b, Supplementary Table 5).

### Weight changes

**Fig. 5:**
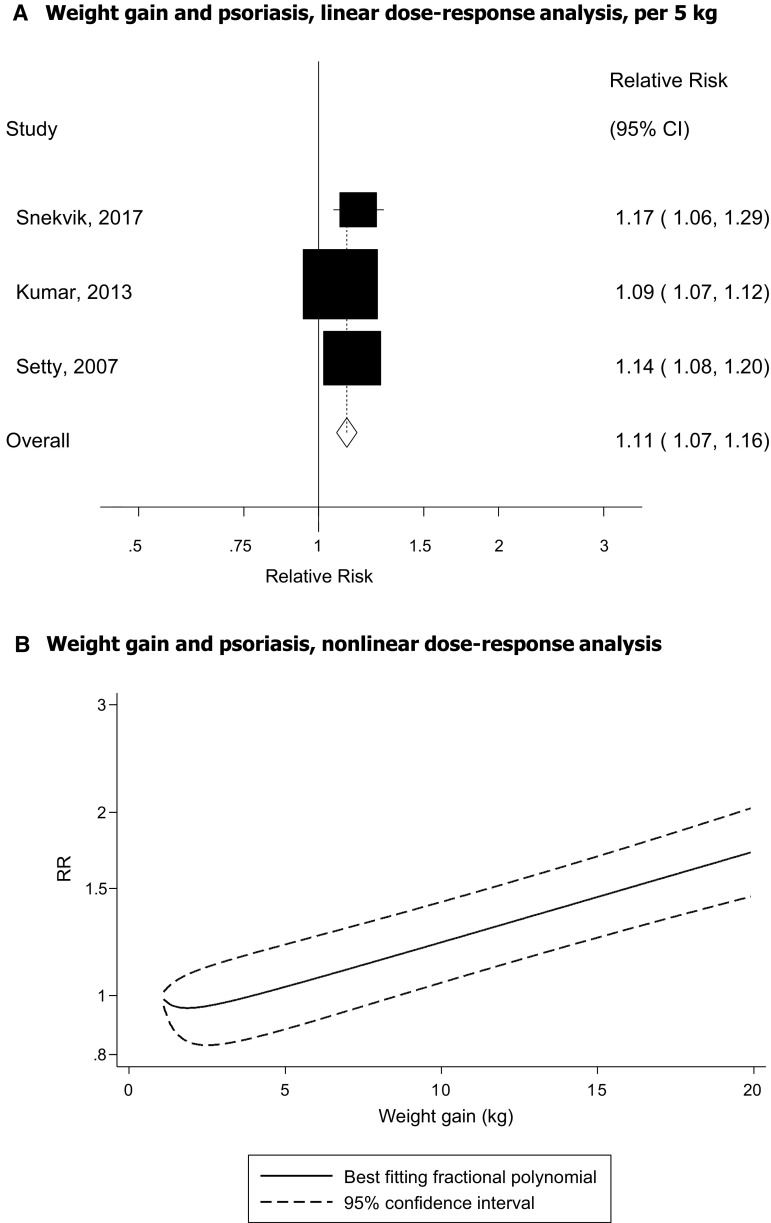
Weight gain and psoriasis

Three cohort studies [44, 45, 49] were included in the analysis of weight changes (between age 18–20 and baseline in the Nurses’ Health Studies I and II and between baseline of HUNT 1 and baseline of HUNT 2) and psoriasis risk and included 1968 cases among 171 074 participants. The summary RR per 5 kg of weight gain was 1.11 (95% CI 1.07–1.16, I^2^ = 46.8%, *p* = 0.15) (Fig. 5a). The summary RR was 1.10 (95% CI 1.07–1.14, I^2^ = 19%, n = 3) for women and 1.27 (95% CI 1.08–1.48, n = 1) for men. There was no evidence of a nonlinear association between weight gain and psoriasis risk, *p*_nonlinearity_ = 0.26 (Fig. 5b, Supplementary Table 6). It was not possible to conduct dose–response analyses of weight loss as all studies only reported risk estimates for one category of weight loss, however, the summary RR comparing weight loss with stable weight was 0.89 (95% CI 0.69–1.13, I^2^ = 0%, *p* = 0.50).

### Subgroup analyses and study quality

In subgroup analyses, there was little evidence of heterogeneity between subgroups when stratified by sex, geographic location, number of cases, and adjustment for confounding factors including age, smoking, alcohol, and physical activity (*p*_heterogeneity_ ≥ 0.07 for all comparisons). There was some evidence of heterogeneity when studies were stratified by duration of follow-up, *p*_heterogeneity_ = 0.03, with a stronger association among studies with a longer duration of follow-up compared to a shorter duration of follow-up (≥ 10 vs. < 10 years follow-up).

The mean (median) study quality scores were 7.3 (7.0) out of 9 possible points for the seven studies included in the dose–response analysis of BMI and psoriasis and five studies had a score of 7 and two studies had a score of 8 points (Table 1).

## Discussion

In this meta-analysis of prospective studies we found evidence of an increased risk of psoriasis with higher BMI, waist circumference, waist-to-hip ratio, and weight gain. There was a 19, 24, 37, and 11% increase in the relative risk of psoriasis for each 5 unit increment in BMI, 10 cm increase in waist circumference, 0.1 unit increment in waist-to-hip ratio and 5 kg of weight gain, respectively. Although there was some evidence of nonlinearity of the association between BMI and psoriasis, there was a clear dose–response relationship, with the lowest risk observed around a BMI of 20, and with a significant increase from a BMI around 22.5–24, and risk increased exponentially with increasing BMI. The associations between waist circumference, waist-to-hip ratio, and weight gain and psoriasis appeared to be linear. The findings are consistent with a previous meta-analysis of cross-sectional and case–control studies which found an increased risk with obesity [43], however, to our knowledge this is the first meta-analysis of only prospective studies to find an increased risk, and the first meta-analysis to investigate the dose–response relationship between different measures of adiposity such as abdominal adiposity and weight gain and psoriasis risk. The previous meta-analysis reported a 66% increase in the relative risk among obese subjects compared to normal weight subjects [43], while we found a 2–4 fold increase in the risk of psoriasis among those at the high end of each adiposity measure compared to those who were slim.

Several potential mechanisms may explain an association between greater adiposity and increased psoriasis risk. Adiposity is associated with chronic, low-grade inflammation through overproduction of inflammatory cytokines. Activated macrophages in adipose tissue stimulate adipocytes to secrete inflammatory mediators such as TNF-α, IL-1, IL-6, and IL-8 which may account for some of the pathologic changes observed in psoriasis patients [23, 24]. Studies in psoriasis patients have also found higher levels of leptin [25–27], an adipokine that is positively correlated to obesity. In addition a positive correlation between leptin and the severity of psoriasis has been observed [25], while lower levels of adiponectin [27, 63] have been found among psoriasis patients. Leptin deficiency has been shown to counteract psoriasis-like skin inflammation in a mouse model, while leptin stimulation of human kerotinocytes has shown to increase the proliferation and to induce secretion of several pro-inflammatory proteins, two of the characteristics of psoriasis [64]. Further support for an important role of obesity in the etiology of psoriasis comes from the observation that weight loss induced by diet and lifestyle changes or obesity surgery in obese psoriasis patients has been found to lead to improvement or remission of the condition over time [65–74]. A diet and exercise intervention was also found to reduce concentrations of TNF-α, IL-6, IL-8, C-reactive protein and monocyte chemoattractant protein 1 [75], which may contribute to improvement of psoriasis. Although we did not find a significant association between weight loss and reduced psoriasis risk in the current analysis, we were not able to conduct dose–response analyses of weight loss and psoriasis risk because the data were reported in only two categories. In addition, the weight loss analysis was based on the total population (including those who were normal weight at baseline) so more pronounced associations may have been observed if those analyses had been restricted to those who were overweight and/or obese at baseline.

Our meta-analysis has some limitations which may affect the interpretation of the results. The main limitation is the low number of prospective studies available reporting on waist circumference, waist-to-hip ratio, and weight gain which limited our possibility to conduct subgroup analyses and test for publication bias for these measures and only one study reported on hip circumference. Although it is possible that confounding may have affected the results as overweight and obese persons usually are less physically active and have unhealthier diets than normal weight persons, it is unlikely that such confounding could entirely explain the association because the risk associated with body fatness is much stronger than those observed for both physical activity [21] and dietary factors [28]. In addition, the results persisted in subgroup analyses by adjustment for confounding factors and there was little evidence of heterogeneity between these subgroups. Nevertheless, the possibility that unidentified risk factors could confound the associations cannot be entirely excluded. There was some evidence of heterogeneity in the subgroup analysis stratified by duration of follow-up, with a stronger association among studies with a longer duration of follow-up compared to a shorter follow-up. It is possible that this to some degree also could reflect weight gain over time as we found a positive association between weight gain and psoriasis and because most of the studies only used the baseline assessment for the analysis of BMI and psoriasis. Two studies also found a stronger association between updated BMI and psoriasis than with baseline BMI [44, 45], which also might suggest that weight gain over time could have led to the observed stronger association among studies with longer follow-up.

Measurement errors in the assessment of height and weight may have influenced our results. Although most of the studies relied on self-reported height and weight, there is generally a high correlation between self-reported and measured height and weight and waist and hip circumferences [76, 77]. In addition, the results were similar when studies were stratified by whether the anthropometric measurements were measured or self-reported. Although meta-analyses of published literature may be susceptible to publication bias, we found no evidence of publication bias with either Egger’s test or with Begg’s test or when visually inspecting the funnel plots, however, we may have had limited power to detect such bias because of the moderate number of studies, and we were not able to reliably test for publication bias in the analyses of waist circumference, waist-to-hip ratio and weight changes.

The assessment of psoriasis diagnoses was based on self-report or validated self-report [44, 45, 47, 49] in some studies and through linkages to medical records or registries in the remaining studies [17, 46, 48], however, several of these studies validated the self-reported diagnoses and found relatively high positive predictive values of between 78% [3], 82% [17] and 92% [45]. While some cases of psoriasis may go undiagnosed, any misclassification of the outcome would tend to attenuate the observed risk estimates because of the prospective nature of the included studies.

Our meta-analysis also has several strengths. Because we based our analysis on prospective studies ambiguity with regard to the temporality of the associations is avoided, and in addition recall bias is not likely to explain our findings, and there is also less possibility for selection bias. In addition, our meta-analysis included large cohort studies with relatively long follow-up and included 17,636 cases and 695 471 participants in the BMI analysis, so we had statistical power to detect even moderate associations. The results were robust to the influence of single studies. The current findings have important public health implications as the prevalence of overweight and obesity has increased globally over the last decades [78], and this could contribute to an increased incidence of psoriasis as well as a range of other chronic diseases over time. In a recent Norwegian study approximately 25% of psoriasis cases could be attributed to overweight and obesity suggesting an important public health impact of excess weight on psoriasis risk [49].

In summary, our meta-analysis confirms a positive association between body fatness, waist circumference, waist-to-hip ratio, and weight gain and psoriasis risk. Any further studies should further assess the association between abdominal obesity and weight changes and psoriasis risk. Our findings confirm the previous recommendations to be as lean as possible within the normal BMI range and suggest that avoiding excess weight gain in adulthood may reduce the risk of psoriasis.

## Electronic supplementary material

Below is the link to the electronic supplementary material.

Supplementary material 1 (DOCX 56 kb)
