# Supplementary material for: Body mass index, abdominal fatness, weight gain and the risk of psoriasis: a systematic review and dose–response meta-analysis of prospective studies
Source: Eur J Epidemiol. 2018 Apr 21;33(12):1163–78. doi: 10.1007/s10654-018-0366-z (PMC6290660; doi:10.1007/s10654-018-0366-z)
Supplement: Supplementary file 1 — Supplementary material 1 (DOCX 56 kb) [file 10654_2018_366_MOESM1_ESM.docx]

Supplementary Text. Search terms used for the PubMed search.

(“body mass index” OR BMI OR overweight OR obesity OR anthropometry OR fatness OR “body fatness” OR “abdominal fatness” OR “abdominal obesity” OR “waist circumference” OR “waist-to-hip ratio” OR adiposity OR "weight gain" OR "weight change" OR “weight loss”) AND psoriasis

Supplementary Text. Search terms used for the Embase search

(body mass index/ OR BMI/ OR overweight/ OR obesity/ OR anthropometry/ OR fatness/ OR body fatness/ OR abdominal fatness/ OR abdominal obesity/ OR waist circumference/ OR waist-to-hip ratio/ OR adiposity/ OR weight gain/ OR weight change/ OR weight loss/ OR (body mass index or BMI or overweight or obesity or anthropometry or fatness or body fatness or abdominal fatness or abdominal obesity or waist circumference or waist-to-hip ratio or adiposity or weight gain or weight change or weight loss).ab,ti ) AND (psoriasis/ OR psoriasis.ab,ti.)

Supplementary Table 1. List of excluded studies and exclusion reason

| Exclusion reason | Reference number |
| --- | --- |
| <3 categories of exposure | (1) |
| Abstract only publication | (2-4) |
| Adolescent BMI | (5) |
| Case-control study | (6-39) |
| Case only study | (40-43) |
| Case report | (44) |
| Childhood psoriasis | (45-47) |
| Commentary, editorial, letter | (48-60) |
| Cross-sectional study | (61-90) |
| Duplicates | (91;92) |
| Meta-analysis | (93-96) |
| No risk estimates | (97;98) |
| Not relevant data | (99) |
| Not relevant exposure | (100;101) |
| Not relevant outcome | (102-104) |
| Obesity diagnosis | (105) |
| Patient populations | (106-109) |
| Review | (110-140) |

Reference List

(1) Vessey MP, Painter R, Powell J. Skin disorders in relation to oral contraception and other factors, including age, social class, smoking and body mass index. Findings in a large cohort study. Br J Dermatol 2000 Oct;143(4):815-20.

(2) Kumar S, Han J, Qureshi A. Obesity, waist circumference, weight change, and the risk of psoriatic arthritis in US women. Journal of Investigative Dermatology Conference: 2011;(var.pagings):April.

(3) Kumar S, Qureshi A, Han J. Obesity, waist circumference, weight change, and the risk of psoriasis (PS) and psoriatic arthritis (PsA) in US women. Journal of the American Academy of Dermatology Conference: 69th Annual Meeting of the American Academy of Dermatology New Orleans, LA United States Conference Publication: (var pagings) 64 (2 SUPPL 1) (pp AB145), 2011;February.

(4) Frankel HC, Han J, Li T, Qureshi AA. Psoriasis and risk of hypertension in US women. Journal of Investigative Dermatology Conference: 2011;(var.pagings):April.

(5) Bryld LE, Sorensen TI, Andersen KK, Jemec GB, Baker JL. High body mass index in adolescent girls precedes psoriasis hospitalization. Acta Derm Venereol 2010 Sep;90(5):488-93.

(6) Albareda M, Ravella A, Castello M, Saborit S, Peramiquel L, Vila L. Metabolic syndrome and its components in patients with psoriasis. Springerplus 2014;3:612.

(7) Duarte GV, Silva LP. Correlation between psoriasis' severity and waist-to-height ratio. An Bras Dermatol 2014 Sep;89(5):846-7.

(8) Menegon DB, Pereira AG, Camerin AC, Cestari T. Psoriasis and comorbidities in a southern Brazilian population: a case-control study. Int J Dermatol 2014 Nov;53(11):e518-e525.

(9) Ali NM, Kuruvila M, Unnikrishnan B. Psoriasis and metabolic syndrome: a case control study. Indian J Dermatol Venereol Leprol 2014 May;80(3):255-7.

(10) Koku Aksu AE, Saracoglu ZN, Metintas S, Sabuncu I, Cetin Y. Age and gender differences in Framingham risk score and metabolic syndrome in psoriasis patients: A cross-sectional study in the Turkish population. Anatol J Cardiol 2016 Apr 25.

(11) Naito R, Imafuku S. Distinguishing features of body mass index and psoriasis in men and women in Japan: A hospital-based case-control study. J Dermatol 2016 May 14.

(12) Naldi L, Parazzini F, Peli L, Chatenoud L, Cainelli T. Dietary factors and the risk of psoriasis. Results of an Italian case-control study. Br J Dermatol 1996 Jan;134(1):101-6.

(13) Herron MD, Hinckley M, Hoffman MS, Papenfuss J, Hansen CB, Callis KP, Krueger GG. Impact of obesity and smoking on psoriasis presentation and management. Arch Dermatol 2005 Dec;141(12):1527-34.

(14) Naldi L, Chatenoud L, Linder D, Belloni FA, Peserico A, Virgili AR, Bruni PL, Ingordo V, Lo SG, Solaroli C, Schena D, Barba A, et al. Cigarette smoking, body mass index, and stressful life events as risk factors for psoriasis: results from an Italian case-control study. J Invest Dermatol 2005 Jul;125(1):61-7.

(15) Sommer DM, Jenisch S, Suchan M, Christophers E, Weichenthal M. Increased prevalence of the metabolic syndrome in patients with moderate to severe psoriasis. Arch Dermatol Res 2006 Dec;298(7):321-8.

(16) Gisondi P, Tessari G, Conti A, Piaserico S, Schianchi S, Peserico A, Giannetti A, Girolomoni G. Prevalence of metabolic syndrome in patients with psoriasis: a hospital-based case-control study. Br J Dermatol 2007 Jul;157(1):68-73.

(17) Jin Y, Zhang F, Yang S, Kong Y, Xiao F, Hou Y, Fan X, Zhang X. Combined effects of HLA-Cw6, body mass index and waist-hip ratio on psoriasis vulgaris in Chinese Han population. J Dermatol Sci 2008 Nov;52(2):123-9.

(18) Wolk K, Mallbris L, Larsson P, Rosenblad A, Vingard E, Stahle M. Excessive body weight and smoking associates with a high risk of onset of plaque psoriasis. Acta Derm Venereol 2009;89(5):492-7.

(19) Xiao J, Chen LH, Tu YT, Deng XH, Tao J. Prevalence of myocardial infarction in patients with psoriasis in central China. J Eur Acad Dermatol Venereol 2009 Nov;23(11):1311-5.

(20) Wolkenstein P, Revuz J, Roujeau JC, Bonnelye G, Grob JJ, Bastuji-Garin S. Psoriasis in France and associated risk factors: results of a case-control study based on a large community survey. Dermatology 2009;218(2):103-9.

(21) Murray ML, Bergstresser PR, ms-Huet B, Cohen JB. Relationship of psoriasis severity to obesity using same-gender siblings as controls for obesity. Clin Exp Dermatol 2009 Mar;34(2):140-4.

(22) Bandoli G, Johnson DL, Jones KL, Lopez JJ, Salas E, Mirrasoul N, Van Voorhees AS, Chambers CD. Potentially modifiable risk factors for adverse pregnancy outcomes in women with psoriasis. Br J Dermatol 2010 Aug;163(2):334-9.

(23) Jensen PR, Zachariae C, Hansen P, Skov L. Normal endothelial function in patients with mild-to-moderate psoriasis: a case-control study. Acta Derm Venereol 2011 Sep;91(5):516-20.

(24) Mebazaa A, El AM, Zidi W, Zayani Y, Cheikh RR, El OS, Kanoun F, Mokni M, Osman AB, Feki M, Slimane H, Mebazaa A, et al. Metabolic syndrome in Tunisian psoriatic patients: prevalence and determinants. J Eur Acad Dermatol Venereol 2011 Jun;25(6):705-9.

(25) Tobin AM, Hughes R, Hand EB, Leong T, Graham IM, Kirby B. Homocysteine status and cardiovascular risk factors in patients with psoriasis: a case-control study. Clin Exp Dermatol 2011 Jan;36(1):19-23.

(26) Zhang C, Zhu KJ, Zheng HF, Cui Y, Zhou FS, Chen YL, Tang XF, Li M, Zhang FY, Fan X, Zuo XB, Yang S, et al. The effect of overweight and obesity on psoriasis patients in Chinese Han population: a hospital-based study. J Eur Acad Dermatol Venereol 2011 Jan;25(1):87-91.

(27) Shapiro J, Cohen AD, Weitzman D, Tal R, David M. Psoriasis and cardiovascular risk factors: a case-control study on inpatients comparing psoriasis to dermatitis. J Am Acad Dermatol 2012 Feb;66(2):252-8.

(28) Damevska K, Neloska L, Gocev G, Mihova M. Metabolic syndrome in untreated patients with psoriasis: case-control study. J Dtsch Dermatol Ges 2013 Dec;11(12):1169-75.

(29) Topic I, Simic D. Prevalence of metabolic syndrome in patients with psoriasis at Mostar Clinical Hospital. Acta Clin Croat 2013 Mar;52(1):53-8.

(30) Tseng HW, Lin HS, Lam HC. Co-morbidities in psoriasis: a hospital-based case-control study. J Eur Acad Dermatol Venereol 2013 Nov;27(11):1417-25.

(31) Kokpol C, Aekplakorn W, Rajatanavin N. Prevalence and characteristics of metabolic syndrome in South-East Asian psoriatic patients: a case-control study. J Dermatol 2014 Oct;41(10):898-902.

(32) Votrubova J, Juzlova K, Smerhovsky Z, Fialova J, Gopfertova D, Vojackova N, Hercogova J. Risk factors for comorbidities in Czech psoriatic patients: results of a hospital-based case-control study. Biomed Pap Med Fac Univ Palacky Olomouc Czech Repub 2014 Jun;158(2):288-94.

(33) Lee A, Smith SD, Hong E, Garnett S, Fischer G. Association Between Pediatric Psoriasis and Waist-to-Height Ratio in the Absence of Obesity: A Multicenter Australian Study. JAMA Dermatol 2016 Sep 28.

(34) Komorowska O, Bohdan M, Szczerkowska-Dobosz A, Rawicz-Zegrzda D, Dudziak M, Zdrojewski T, Gruchala M, Purzycka-Bohdan D, Nowicki R. Assessment of Cardiovascular Risk Factors in Patients with Psoriasis. Acta Dermatovenerol Croat 2016 Dec;24(4):261-7.

(35) Girisha BS, Thomas N. Metabolic Syndrome in Psoriasis among Urban South Indians: A Case Control Study Using SAM-NCEP Criteria. J Clin Diagn Res 2017 Feb;11(2):WC01-WC04.

(36) Ozden MG, Tekin NS, Gurer MA, Akdemir D, Dogramaci C, Utas S, Akman A, Evans SE, Bahadir S, Ozturkcan S, Ikizoglu G, Sendur N, et al. Environmental risk factors in pediatric psoriasis: a multicenter case-control study. Pediatr Dermatol 2011 May;28(3):306-12.

(37) Bhole VM, Choi HK, Burns LC, Vera KC, Lacaille DV, Gladman DD, Dutz JP. Differences in body mass index among individuals with PsA, psoriasis, RA and the general population. Rheumatology (Oxford) 2012 Mar;51(3):552-6.

(38) Mahe E, Beauchet A, Bodemer C, Phan A, Bursztejn AC, Boralevi F, Souillet AL, Chiaverini C, Bourrat E, Miquel J, Vabres P, Barbarot S, et al. Psoriasis and obesity in French children: a case-control, multicentre study. Br J Dermatol 2015 Jun;172(6):1593-600.

(39) Diniz MS, Bavoso NC, Kakehasi AM, Lauria MW, Soares MM, hado-Pinto J. Assessment of adiposity in psoriatic patients by dual energy X-ray absorptiometry compared to conventional methods. An Bras Dermatol 2016 Apr;91(2):150-5.

(40) Marino MG, Carboni I, De FC, Maurici M, Maccari F, Franco E. Risk factors for psoriasis: a retrospective study on 501 outpatients clinical records. Ann Ig 2004 Nov;16(6):753-8.

(41) Bardazzi F, Balestri R, Baldi E, Antonucci A, De TS, Patrizi A. Correlation between BMI and PASI in patients affected by moderate to severe psoriasis undergoing biological therapy. Dermatol Ther 2010 Jan;23 Suppl 1:S14-S19.

(42) Chularojanamontri L, Wongpraparut C, Silpa-Archa N, Chaweekulrat P. Metabolic syndrome and psoriasis severity in South-East Asian patients: An investigation of potential association using current and chronological assessments. J Dermatol 2016 Aug 11.

(43) Heredi E, Csordas A, Clemens M, Adam B, Gaspar K, Torocsik D, Nagy G, Adany R, Gaal J, Remenyik E, Szegedi A. The prevalence of obesity is increased in patients with late compared with early onset psoriasis. Ann Epidemiol 2013 Nov;23(11):688-92.

(44) Nowlin N, Solomon H. Letter: Weight loss and psoriasis. Arch Dermatol 1976 Oct;112(10):1465.

(45) Boccardi D, Menni S, La VC, Nobile M, Decarli A, Volpi G, Ferraroni M. Overweight and childhood psoriasis. Br J Dermatol 2009 Aug;161(2):484-6.

(46) Zhu KJ, He SM, Zhang C, Yang S, Zhang XJ. Relationship of the body mass index and childhood psoriasis in a Chinese Han population: a hospital-based study. J Dermatol 2012 Feb;39(2):181-3.

(47) Becker L, Tom WL, Eshagh K, Benjamin LT, Paller AS. Excess adiposity preceding pediatric psoriasis. JAMA Dermatol 2014 May;150(5):573-4.

(48) Larios G, Alevizos A, Rigopoulos D. Risk of psoriasis and obesity in women. Arch Intern Med 2008 Mar 24;168(6):666.

(49) Naldi L. Should we use body mass index to predict disease onset and severity in psoriasis? Acta Derm Venereol 2009;89(5):452.

(50) Girolomoni G, Gisondi P. Psoriasis and metabolic comorbidities: The importance of well-designed prospective studies. Dermatology 216 (2) (pp 130-132), 2008;January.

(51) Dellavalle RP, Johnson KR. Do smoking, obesity, and stress cause psoriasis? J Invest Dermatol 2005 Jul;125(1):vi-vii.

(52) Weinberg JM. Lifestyle issues and psoriasis. Cutis 2006 Sep;78(3):160.

(53) Lebwhol M, Callen JP. Obesity, smoking, and psoriasis. JAMA 2006 Jan 11;295(2):208-10.

(54) Caglia MT, Krueger GG. Psoriasis and the obesity epidemic: the effect of weight loss. JAMA Dermatol 2013 Jul;149(7):786-7.

(55) Gelfand JM. Psoriasis, type 2 diabetes mellitus, and obesity weighing the evidence. JAMA Dermatology 152 (7) (pp 753-754), 2016;July.

(56) Forman L. Psoriasis and obesity. British Journal of Dermatology 89 (1) (pp 99), 1973;1973.

(57) McGowan JW, Pearce DJ, Chen J, Richmond D, Balkrishnan R, Feldman SR. The skinny on psoriasis and obesity. Arch Dermatol 2005 Dec;141(12):1601-2.

(58) Perez-Perez L, Allegue F, Caeiro JL, Zulaica JM. Severe psoriasis, morbid obesity and bariatric surgery. Clin Exp Dermatol 2009 Oct;34(7):e421-e422.

(59) Hossler EW, Wood GC, Still CD, Mowad CM, Maroon MS. The effect of weight loss surgery on the severity of psoriasis. Br J Dermatol 2013 Mar;168(3):660-1.

(60) Tobin AM, Hackett CB, Rogers S, Collins P, Richards HL, O'Shea D, Kirby B. Body mass index, waist circumference and HOMA-IR correlate with the Psoriasis Area and Severity Index in patients with psoriasis receiving phototherapy. British Journal of Dermatology 171 (2) (pp 436-438), 2014;2014.

(61) Helmick CG, Lee-Han H, Hirsch SC, Baird TL, Bartlett CL. Prevalence of psoriasis among adults in the U.S.: 2003-2006 and 2009-2010 National Health and Nutrition Examination Surveys. Am J Prev Med 2014 Jul;47(1):37-45.

(62) Miller IM, Ellervik C, Zarchi K, Ibler KS, Vinding GR, Knudsen KM, Jemec GB. The association of metabolic syndrome and psoriasis: a population- and hospital-based cross-sectional study. J Eur Acad Dermatol Venereol 2015 Mar;29(3):490-7.

(63) Lonnberg AS, Skov L, Skytthe A, Kyvik KO, Pedersen OB, Thomsen SF. Association of Psoriasis With the Risk for Type 2 Diabetes Mellitus and Obesity. JAMA Dermatol 2016 Jul 1;152(7):761-7.

(64) Kimball AB, Robinson D, Jr., Wu Y, Guzzo C, Yeilding N, Paramore C, Fraeman K, Bala M. Cardiovascular disease and risk factors among psoriasis patients in two US healthcare databases, 2001-2002. Dermatology 2008;217(1):27-37.

(65) Cohen AD, Sherf M, Vidavsky L, Vardy DA, Shapiro J, Meyerovitch J. Association between psoriasis and the metabolic syndrome. A cross-sectional study. Dermatology 2008;216(2):152-5.

(66) Takahashi H, Tsuji H, Takahashi I, Hashimoto Y, Ishida-Yamamoto A, Iizuka H. Prevalence of obesity/adiposity in Japanese psoriasis patients: adiposity is correlated with the severity of psoriasis. J Dermatol Sci 2009 Jul;55(1):74-6.

(67) Zingone F, Bucci C, Tortora R, Santonicola A, Cappello C, Franzese MD, Passananti V, Ciacci C. Body mass index and prevalence of skin diseases in adults with untreated coeliac disease. Digestion 2009;80(1):18-24.

(68) Takahashi H, Tsuji H, Takahashi I, Hashimoto Y, Ishida-Yamamoto A, Iizuka H. Prevalence of obesity/adiposity in Japanese psoriasis patients: adiposity is correlated with the severity of psoriasis. J Dermatol Sci 2009 Apr;54(1):61-3.

(69) Vena GA, Altomare G, Ayala F, Berardesca E, Calzavara-Pinton P, Chimenti S, Giannetti A, Girolomoni G, Lotti T, Martini P, Mazzaglia G, Peserico A, et al. Incidence of psoriasis and association with comorbidities in Italy: a 5-year observational study from a national primary care database. Eur J Dermatol 2010 Sep;20(5):593-8.

(70) Huang YH, Yang LC, Hui RY, Chang YC, Yang YW, Yang CH, Chen YH, Chung WH, Kuan YZ, Chiu CS. Relationships between obesity and the clinical severity of psoriasis in Taiwan. J Eur Acad Dermatol Venereol 2010 Sep;24(9):1035-9.

(71) Augustin M, Glaeske G, Radtke MA, Christophers E, Reich K, Schafer I. Epidemiology and comorbidity of psoriasis in children. Br J Dermatol 2010 Mar;162(3):633-6.

(72) Bongiorno MR, Doukaki S, Rizzo D, Arico M. The prevalence of the obesity in patients with moderate to severe psoriasis in Sicily populations. J Eur Acad Dermatol Venereol 2010 Jan;24(1):92-3.

(73) Warnecke C, Manousaridis I, Herr R, Terris DD, Goebeler M, Goerdt S, Peitsch WK. Cardiovascular and metabolic risk profile in German patients with moderate and severe psoriasis: a case control study. Eur J Dermatol 2011 Sep;21(5):761-70.

(74) Koebnick C, Black MH, Smith N, Der-Sarkissian JK, Porter AH, Jacobsen SJ, Wu JJ. The association of psoriasis and elevated blood lipids in overweight and obese children. J Pediatr 2011 Oct;159(4):577-83.

(75) Islam MT, Paul HK, Zakaria SM, Islam MM, Shafiquzzaman M. Epidemiological determinants of psoriasis. Mymensingh Med J 2011 Jan;20(1):9-15.

(76) Love TJ, Qureshi AA, Karlson EW, Gelfand JM, Choi HK. Prevalence of the metabolic syndrome in psoriasis: results from the National Health and Nutrition Examination Survey, 2003-2006. Arch Dermatol 2011 Apr;147(4):419-24.

(77) Langan SM, Seminara NM, Shin DB, Troxel AB, Kimmel SE, Mehta NN, Margolis DJ, Gelfand JM. Prevalence of metabolic syndrome in patients with psoriasis: a population-based study in the United Kingdom. J Invest Dermatol 2012 Mar;132(3 Pt 1):556-62.

(78) Santos M, Fonseca HM, Jalkh AP, Gomes GP, Cavalcante AS. Obesity and dyslipidemia in patients with psoriasis treated at a dermatologic clinic in Manaus. An Bras Dermatol 2013 Nov;88(6):913-6.

(79) Kim CR, Lee JH. An observational study on the obesity and metabolic status of psoriasis patients. Ann Dermatol 2013 Nov;25(4):440-4.

(80) Danielsen K, Olsen AO, Wilsgaard T, Furberg AS. Is the prevalence of psoriasis increasing? A 30-year follow-up of a population-based cohort. Br J Dermatol 2013 Jun;168(6):1303-10.

(81) Duarte GV, Oliveira MF, Cardoso TM, Follador I, Silva TS, Cavalheiro CM, Nonato W, Carvalho EM. Association between obesity measured by different parameters and severity of psoriasis. Int J Dermatol 2013 Feb;52(2):177-81.

(82) Casagrande SS, Menke A, Cowie CC. No association between psoriasis and diabetes in the U.S. population. Diabetes Res Clin Pract 2014 Jun;104(3):e58-e60.

(83) Bostoen J, Van PL, Brochez L, Mielants H, Lambert J. A cross-sectional study on the prevalence of metabolic syndrome in psoriasis compared to psoriatic arthritis. J Eur Acad Dermatol Venereol 2014 Apr;28(4):507-11.

(84) Ma L, Li M, Wang H, Li Y, Bai B. High prevalence of cardiovascular risk factors in patients with moderate or severe psoriasis in northern China. Arch Dermatol Res 2014 Apr;306(3):247-51.

(85) Gyldenlove M, Jensen P, Linneberg A, Thyssen JP, Zachariae C, Hansen PR, Skov L. Psoriasis and the Framingham risk score in a Danish hospital cohort. Int J Dermatol 2014 Sep;53(9):1086-90.

(86) Carrascosa JM, Vilavella M, Garcia-Doval I, Carretero G, Vanaclocha F, Dauden E, Gomez-Garcia FJ, Herrera-Ceballos E, De la Cueva DP, Belinchon I, Sanchez-Carazo JL, Alsina M, et al. Body mass index in patients with moderate-to-severe psoriasis in Spain and its impact as an independent risk factor for therapy withdrawal: results of the Biobadaderm Registry. J Eur Acad Dermatol Venereol 2014 Jul;28(7):907-14.

(87) Gui XY, Yu XL, Jin HZ, Zuo YG, Wu C. Prevalence of metabolic syndrome in Chinese psoriasis patients: A hospital-based cross-sectional study. J Diabetes Investig 2017 Mar 30.

(88) Milcic D, Jankovic S, Vesic S, Milinkovic M, Marinkovic J, Cirkovic A, Jankovic J. Prevalence of metabolic syndrome in patients with psoriasis: a hospital-based cross-sectional study. An Bras Dermatol 2017 Jan;92(1):46-51.

(89) Jacobi A, Langenbruch A, Purwins S, Augustin M, Radtke MA. Prevalence of Obesity in Patients with Psoriasis: Results of the National Study PsoHealth3. Dermatology 2015;231(3):231-8.

(90) Danielsen K, Wilsgaard T, Olsen AO, Eggen AE, Olsen K, Cassano PA, Furberg AS. Elevated odds of metabolic syndrome in psoriasis: a population-based study of age and sex differences. Br J Dermatol 2015 Feb;172(2):419-27.

(91) Li WQ, Han JL, Zhang MF, Qureshi AA. Interactions between adiposity and genetic polymorphisms on the risk of psoriasis. Br J Dermatol 2013 Mar;168(3):639-42.

(92) Neimann AL, Shin DB, Wang X, Margolis DJ, Troxel AB, Gelfand JM. Prevalence of cardiovascular risk factors in patients with psoriasis. J Am Acad Dermatol 2006 Nov;55(5):829-35.

(93) Armstrong AW, Harskamp CT, Armstrong EJ. The association between psoriasis and obesity: a systematic review and meta-analysis of observational studies. Nutr Diabetes 2012 Dec 3;2:e54.

(94) Miller IM, Ellervik C, Yazdanyar S, Jemec GB. Meta-analysis of psoriasis, cardiovascular disease, and associated risk factors. J Am Acad Dermatol 2013 Dec;69(6):1014-24.

(95) Gaeta M, Castelvecchio S, Ricci C, Pigatto P, Pellissero G, Cappato R. Role of psoriasis as independent predictor of cardiovascular disease: a meta-regression analysis. Int J Cardiol 2013 Oct 3;168(3):2282-8.

(96) Upala S, Sanguankeo A. Effect of lifestyle weight loss intervention on disease severity in patients with psoriasis: a systematic review and meta-analysis. Int J Obes (Lond) 2015 Aug;39(8):1197-202.

(97) Johnson JA, Ma C, Kanada KN, Armstrong AW. Diet and nutrition in psoriasis: Analysis of the National Health and Nutrition Examination Survey (NHANES) in the United States. Journal of the European Academy of Dermatology and Venereology 28 (3) (pp 327-332), 2014;March.

(98) Yang Y-W, Kang J-H, Lin H-C. Increased risk of psoriasis following obstructive sleep apnea: A longitudinal population-based study. Sleep Medicine (no pagination) 2012;(no pagination).

(99) Radner H, Lesperance T, Accortt NA, Solomon DH. Incidence and Prevalence of Cardiovascular Risk Factors Among Patients With Rheumatoid Arthritis, Psoriasis, or Psoriatic Arthritis. Arthritis Care Res (Hoboken ) 2016 Dec 20.

(100) Frankel HC, Han J, Li T, Qureshi AA. The association between physical activity and the risk of incident psoriasis. Archives of Dermatology 148 (8) (pp 918-924), 2012;August.

(101) Egeberg A, Sorensen JA, Gislason GH, Knop FK, Skov L. Incidence and prognosis of psoriasis and psoriatic arthritis in patients undergoing bariatric surgery. JAMA Surgery 152 (4) (pp 344-349), 2017;April.

(102) Love TJ, Zhu Y, Zhang Y, Wall-Burns L, Ogdie A, Gelfand JM, Choi HK. Obesity and the risk of psoriatic arthritis: A population-based study. Annals of the Rheumatic Diseases 71 (8) (pp 1273-1277), 2012;August.

(103) Li W, Han J, Qureshi AA. Obesity and risk of incident psoriatic arthritis in US women. Annals of the Rheumatic Diseases 71 (8) (pp 1267-1272), 2012;August.

(104) Ozkaya DB, Onsun N, Topukcu B, Su O, Bahali AG, Dizman D, Rezvani A, Uysal O. The relationship between body mass index, waist circumference and psoriatic arthritis in the Turkish population. Postepy Dermatologii i Alergologii 33 (3) (pp 219-223), 2016;June.

(105) Hemminki K, Li X, Sundquist J, Sundquist K. Risk of asthma and autoimmune diseases and related conditions in patients hospitalized for obesity. Ann Med 2012 May;44(3):289-95.

(106) Jensen P, Zachariae C, Christensen R, Geiker NR, Schaadt BK, Stender S, Hansen PR, Astrup A, Skov L. Effect of weight loss on the severity of psoriasis: a randomized clinical study. JAMA Dermatol 2013 Jul;149(7):795-801.

(107) Naldi L, Addis A, Chimenti S, Giannetti A, Picardo M, Tomino C, Maccarone M, Chatenoud L, Bertuccio P, Caggese E, Cuscito R. Impact of body mass index and obesity on clinical response to systemic treatment for psoriasis. Evidence from the Psocare project. Dermatology 2008;217(4):365-73.

(108) Del GM, Gisondi P, Tessari G, Girolomoni G. Weight reduction alone may not be sufficient to maintain disease remission in obese patients with psoriasis: a randomized, investigator-blinded study. Dermatology 2012;224(1):31-7.

(109) Jensen P, Christensen R, Zachariae C, Geiker NR, Schaadt BK, Stender S, Hansen PR, Astrup A, Skov L. Long-term effects of weight reduction on the severity of psoriasis in a cohort derived from a randomized trial: a prospective observational follow-up study. Am J Clin Nutr 2016 Aug;104(2):259-65.

(110) Mallbris L, Ritchlin CT, Stahle M. Metabolic disorders in patients with psoriasis and psoriatic arthritis. Curr Rheumatol Rep 2006 Oct;8(5):355-63.

(111) Sterry W, Strober BE, Menter A. Obesity in psoriasis: the metabolic, clinical and therapeutic implications. Report of an interdisciplinary conference and review. Br J Dermatol 2007 Oct;157(4):649-55.

(112) Yosipovitch G, DeVore A, Dawn A. Obesity and the skin: skin physiology and skin manifestations of obesity. J Am Acad Dermatol 2007 Jun;56(6):901-16.

(113) Azfar RS, Gelfand JM. Psoriasis and metabolic disease: epidemiology and pathophysiology. Curr Opin Rheumatol 2008 Jul;20(4):416-22.

(114) Ortonne JP. [Psoriasis, metabolic syndrome and its components]. Ann Dermatol Venereol 2008 Feb;135 Suppl 4:S235-S242.

(115) Gottlieb AB, Chao C, Dann F. Psoriasis comorbidities. J Dermatolog Treat 2008;19(1):5-21.

(116) Gisondi P, Girolomoni G. Psoriasis and atherothrombotic diseases: Disease-specific and non-disease-specific risk factors. Seminars in Thrombosis and Hemostasis 35 (3) (pp 313-324), 2009;April.

(117) Gisondi P, Ferrazzi A, Girolomoni G. Metabolic comorbidities and psoriasis. Acta Dermatovenerol Croat 2010;18(4):297-304.

(118) Bremmer S, Van Voorhees AS, Hsu S, Korman NJ, Lebwohl MG, Young M, Bebo BF, Jr., Blauvelt A. Obesity and psoriasis: from the Medical Board of the National Psoriasis Foundation. J Am Acad Dermatol 2010 Dec;63(6):1058-69.

(119) Duarte GV, Follador I, Cavalheiro CM, Silva TS, Oliveira MF. Psoriasis and obesity: literature review and recommendations for management. An Bras Dermatol 2010 May;85(3):355-60.

(120) Davidovici BB, Sattar N, Prinz J, Puig L, Emery P, Barker JN, van de KP, Stahle M, Nestle FO, Girolomoni G, Krueger JG. Psoriasis and systemic inflammatory diseases: potential mechanistic links between skin disease and co-morbid conditions. J Invest Dermatol 2010 Jul;130(7):1785-96.

(121) Kim N, Thrash B, Menter A. Comorbidities in psoriasis patients. Semin Cutan Med Surg 2010 Mar;29(1):10-5.

(122) Gisondi P, Del GM, Cozzi A, Girolomoni G. Psoriasis, the liver, and the gastrointestinal tract. Dermatol Ther 2010 Mar;23(2):155-9.

(123) Hercogova J, Ricceri F, Tripo L, Lotti T, Prignano F. Psoriasis and body mass index. Dermatol Ther 2010 Mar;23(2):152-4.

(124) Alsufyani MA, Golant AK, Lebwohl M. Psoriasis and the metabolic syndrome. Dermatol Ther 2010 Mar;23(2):137-43.

(125) Naldi L, Mercuri SR. Epidemiology of comorbidities in psoriasis. Dermatol Ther 2010 Mar;23(2):114-8.

(126) Menter A, Griffiths CE, Tebbey PW, Horn EJ, Sterry W. Exploring the association between cardiovascular and other disease-related risk factors in the psoriasis population: the need for increased understanding across the medical community. J Eur Acad Dermatol Venereol 2010 Dec;24(12):1371-7.

(127) Shipman AR, Millington GW. Obesity and the skin. Br J Dermatol 2011 Oct;165(4):743-50.

(128) Puig L. Obesity and psoriasis: body weight and body mass index influence the response to biological treatment. J Eur Acad Dermatol Venereol 2011 Sep;25(9):1007-11.

(129) Takahashi H, Iizuka H. Psoriasis and metabolic syndrome. J Dermatol 2012 Mar;39(3):212-8.

(130) Tobin AM, Ahern T, Rogers S, Collins P, O'Shea D, Kirby B. The dermatological consequences of obesity. Int J Dermatol 2013 Aug;52(8):927-32.

(131) Carrascosa JM, Rocamora V, Fernandez-Torres RM, Jimenez-Puya R, Moreno JC, Coll-Puigserver N, Fonseca E. Obesity and psoriasis: inflammatory nature of obesity, relationship between psoriasis and obesity, and therapeutic implications. Actas Dermosifiliogr 2014 Jan;105(1):31-44.

(132) Correia B, Torres T. Obesity: a key component of psoriasis. Acta Biomed 2015 Sep 14;86(2):121-9.

(133) Fleming P, Kraft J, Gulliver WP, Lynde C. The Relationship of Obesity With the Severity of Psoriasis: A Systematic Review. J Cutan Med Surg 2015 Sep;19(5):450-6.

(134) Gutmark-Little I, Shah KN. Obesity and the metabolic syndrome in pediatric psoriasis. Clin Dermatol 2015 May;33(3):305-15.

(135) Barrea L, Nappi F, Di SC, Savanelli MC, Falco A, Balato A, Balato N, Savastano S. Environmental Risk Factors in Psoriasis: The Point of View of the Nutritionist. Int J Environ Res Public Health 2016 Jul 22;13(5).

(136) Jensen P, Skov L. Psoriasis and Obesity. Dermatology 2016;232(6):633-9.

(137) Li W-Q, Cho E, Weinstock MA, Mashfiq H, Qureshi AA. Epidemiological Assessments of Skin Outcomes in the Nurses' Health Studies. American journal of public health 106 (9) (pp 1677-1683), 2016;01.

(138) Colditz GA, Philpott SE, Hankinson SE. The Impact of the Nurses' Health Study on Population Health: Prevention, Translation, and Control. American journal of public health 106 (9) (pp 1540-1545), 2016;01.

(139) Jensen P, Skov L. Psoriasis and Obesity. Dermatology 232 (6) (pp 633-639), 2017;01.

(140) Raychaudhuri SP, Gross J. Psoriasis risk factors: role of lifestyle practices. Cutis 2000 Nov;66(5):348-52.

Supplementary Table 2: Subgroup analyses of BMI and psoriasis

|  | | **BMI** | | | | |
| --- | --- | --- | --- | --- | --- | --- |
|  | | *n* | RR (95% CI) | *I^2^* (%) | *P*_h_^1^ | *P*_h_^2^ |
| All studies | | 7 | 1.19 (1.10-1.28) | 83.1 | <0.0001 |  |
| Duration of follow-up | |  |  |  |  |  |
| <10 yrs follow-up | | 2 | 1.08 (1.01-1.15) | 63.2 | 0.10 | 0.03 |
| ≥10 yrs follow-up | | 5 | 1.25 (1.17-1.33) | 40.4 | 0.15 |  |
| Assessment of weight/height | |  |  |  |  |  |
| Self-reported | | 1 | 1.39 (1.10-1.75) |  |  | 0.07 |
| Self-reported (validated) | | 2 | 1.26 (1.17-1.36) | 52.1 | 0.15 |  |
| Measured | | 4 | 1.12 (1.04-1.21) | 73.2 | 0.01 |  |
| Sex | |  |  |  |  |  |
| Men | | 2 | 1.34 (1.11-1.62) | 35.0 | 0.22 | 0.006/0.41 |
| Women | | 5 | 1.25 (1.19-1.31) | 0.5 | 0.40 |  |
| Men and women | | 2 | 1.08 (1.01-1.15) | 63.2 | 0.10 |  |
| Geographic location | |  |  |  |  |  |
| Europe | | 4 | 1.19 (1.08-1.31) | 59.0 | 0.06 | 0.43 |
| America | | 2 | 1.26 (1.17-1.36) | 52.1 | 0.15 |  |
| Asia | | 1 | 1.05 (1.00-1.10) |  |  |  |
| Number of cases | |  |  |  |  |  |
| Cases <249 | | 1 | 1.39 (1.10-1.75) |  |  | 0.36 |
| Cases 250<500 | | 2 | 1.20 (1.02-1.41) | 68.6 | 0.07 |  |
| Cases ≥500 | | 4 | 1.17 (1.06-1.28) | 89.3 | <0.0001 |  |
| Study quality | |  |  |  |  |  |
| 0-3 | | 0 |  |  |  | NC |
| 4-6 | | 0 |  |  |  |  |
| 7-9 | | 7 | 1.19 (1.10-1.28) | 83.1 | <0.0001 |  |
| Adjustment for confounders | | | | | | |
| Age | Yes | 7 | 1.19 (1.10-1.28) | 83.1 | <0.0001 | NC |
|  | No | 0 |  |  |  |  |
| Alcohol | Yes | 5 | 1.19 (1.07-1.33) | 87.1 | <0.0001 | 0.95 |
|  | No | 2 | 1.19 (1.03-1.38) | 77.4 | 0.04 |  |
| Smoking | Yes | 7 | 1.19 (1.10-1.28) | 83.1 | <0.0001 | NC |
|  | No | 0 |  |  |  |  |
| Physical activity | Yes | 3 | 1.12 (1.00-1.24) | 83.0 | 0.003 | 0.18 |
|  | No | 4 | 1.25 (1.13-1.39) | 76.1 | 0.006 |  |

*n* denotes the number of risk estimates

^1^ P for heterogeneity within each subgroup,

^2^ P for heterogeneity between subgroups with meta-regression analysis

Supplementary Table 3: Body mass index and psoriasis, nonlinear dose-response

| BMI | RR (95% CI) |
| --- | --- |
| 16.75 | 0.98 (0.93-1.03) |
| 17.5 | 0.98 (0.95-1.02) |
| 20 | 1.00 |
| 22 | 1.03 (1.00-1.05) |
| 22.5 | 1.04 (1.01-1.06) |
| 24 | 1.07 (1.02-1.11) |
| 25 | 1.09 (1.04-1.15) |
| 27.5 | 1.18 (1.11-1.25) |
| 30 | 1.30 (1.22-1.38) |
| 32.5 | 1.47 (1.38-1.56) |
| 35 | 1.70 (1.58-1.83) |
| 37.5 | 2.03 (1.83-2.26) |
| 40 | 2.49 (2.12-2.92) |
| 42.5 | 3.17 (2.50-4.02) |
| 45 | 4.15 (2.98-5.79) |
| P_nonlinearity_ | <0.0001 |

Supplementary Table 4: Waist circumference and psoriasis, nonlinear dose-response

| Waist circumference | RR (95% CI) |
| --- | --- |
| 67.41 | 1.00 |
| 70 | 1.02 (0.96-1.08) |
| 75 | 1.09 (0.97-1.23) |
| 80 | 1.22 (1.04-1.43) |
| 85 | 1.39 (1.17-1.64) |
| 90 | 1.61 (1.35-1.92) |
| 95 | 1.87 (1.55-2.26) |
| 100 | 2.19 (1.77-2.71) |
| 105 | 2.56 (2.00-3.29) |
| 110 | 2.99 (2.23-4.02) |
| P_nonlinearity_ | 0.09 |

Supplementary Table 5: Waist-to-hip ratio and psoriasis, nonlinear dose-response

| WHR | RR (95% CI) |
| --- | --- |
| 0.711 | 1.00 |
| 0.750 | 1.08 (0.90-1.30) |
| 0.800 | 1.27 (1.03-1.57) |
| 0.850 | 1.57 (1.29-1.91) |
| 0.900 | 1.99 (1.33-2.97) |
| 0.950 | 2.58 (1.21-5.51) |
| 1.000 | 3.35 (1.03-10.87) |
| P_nonlinearity_ | 0.59 |

Supplementary Table 6: Weight change and psoriasis, nonlinear dose-response

| Weight change (kg) | RR (95% CI) |
| --- | --- |
| +1 | 1.00 |
| +5 | 1.03 (0.87-1.22) |
| +10 | 1.22 (1.04-1.43) |
| +15 | 1.45 (1.23-1.70) |
| +20 | 1.72 (1.44-2.05) |
| P_nonlinearity_ | 0.26 |

Supplementary Figure 1. Funnel plot of BMI and psoriasis
